# Factors determining anti-poliovirus type 3 antibodies among orally immunised Indian infants

**DOI:** 10.1016/j.vaccine.2016.08.032

**Published:** 2016-09-22

**Authors:** Saravanakumar Puthupalayam Kaliappan, Srinivasan Venugopal, Sidhartha Giri, Ira Praharaj, Arun S. Karthikeyan, Sudhir Babji, Jacob John, Jayaprakash Muliyil, Nicholas Grassly, Gagandeep Kang

**Affiliations:** a Division of Gastrointestinal Sciences, Christian Medical College, Vellore, India; b Department of Community Health, Christian Medical College, Vellore, India; c Department of Infectious Disease Epidemiology, Imperial College London, United Kingdom

**Keywords:** tOPV, trivalent oral polio vaccine, PV3, poliovirus serotype 3, WHO, World Health Organization, MLR, multilevel logistic regression, SD, standard deviation, CI, confidence interval, OR, odds ratio, OPV, oral poliovirus vaccine, mOPV3, serotype-3 monovalent oral poliovirus vaccine, IPV, inactivated poliovirus vaccine, HSC, health sub-centre, PHC, primary health centre, SIA, supplemental immunisation activity, CDC, Centers for Disease Control and Prevention, TCID_50_, median tissue culture infective dose, IQR, interquartile range, χ^2^, Chi-square, Poliovirus, Seroprevalence, OPV, Infants, Immunogenicity, India

## Abstract

**Background:**

Among the three poliovirus serotypes, the lowest responses after vaccination with trivalent oral polio vaccine (tOPV) are to serotype 3. Although improvements in routine immunisation and supplementary immunisation activities have greatly increased vaccine coverage, there are limited data on antibody prevalence in Indian infants.

**Methods:**

Children aged 5–11 months with a history of not having received inactivated polio vaccine were screened for serum antibodies to poliovirus serotype 3 (PV3) by a micro-neutralisation assay according to a modified World Health Organization (WHO) protocol. Limited demographic information was collected to assess risk-factors for a lack of protective antibodies. Student’s *t*-test, logistic regression and multilevel logistic regression (MLR) model were used to estimate model parameters.

**Results:**

Of 8454 children screened at a mean age of 8.3 (standard deviation [SD]-1.8) months, 88.1% (95% confidence interval (CI): 87.4–88.8) had protective antibodies to PV3. The number of tOPV doses received was the main determinant of seroprevalence; the maximum likelihood estimate yields a 37.7% (95% CI: 36.2–38.3) increase in seroprevalence per dose of tOPV. In multivariable logistic regression analysis increasing age, male sex, and urban residence were also independently associated with seropositivity (Odds Ratios (OR): 1.17 (95% CI: 1.12–1.23) per month of age, 1.27 (1.11–1.46) and 1.24 (1.05–1.45) respectively).

**Conclusion:**

Seroprevalence of antibodies to PV3 is associated with age, gender and place of residence, in addition to the number of tOPV doses received. Ensuring high coverage and monitoring of response are essential as long as oral vaccines are used in polio eradication.

## Highlights

• 88.1% of 8454 children screened had protective antibodies to poliovirus serotype 3.
• The number of tOPV doses received was the main determinant of seroprevalence.
• Age, gender, residence and number of tOPV doses are associated with seroprevalence.

## 1 Introduction

The global incidence of polio cases has declined with only two countries now considered polio endemic [1]. This remarkable reduction was achieved by the effective use of vaccines, with oral poliovirus vaccines (OPV) playing the greatest role in decreasing disease and interruption of transmission in developing countries. Although OPV has many practical advantages for mass immunisation in field settings [2], like other oral vaccines the immunogenicity and effectiveness of OPV is impaired in lower-income countries [3], [4], [5]. Potential contributing factors for low immunogenicity in these settings include a high prevalence of diarrhoea, infection of the gut with other pathogens, malnutrition, micronutrient deficiencies, and tropical enteropathy [3], [6].

Diarrhoea is independently associated with a failure to seroconvert following administration of OPV after adjusting for potential confounders like season, breast feeding, mass campaigns and maternal antibodies [7]. In northern India, reduced take of OPV was significantly associated with season [8]. Concurrent enteric infections with lower OPV response in low-income settings have been described [9], [10]. Tropical enteropathy, resulting from high environmental exposure to enteric pathogens, is common among children living in poverty and may be associated with poor response to oral vaccines, both in terms of primary antibody response and its longevity [11].

Serological data are informative about vaccination coverage, immunogenicity, secondary spread of vaccine poliovirus and exposure to wild-type infections. However, there are limited published data available on antibodies to polio in Indian children in the recent past, particularly from southern India. Table 1 presents a comparison of recent data on seroprevalence from developing countries.

After vaccination with tOPV, antibody responses are greatest to poliovirus type 2 and usually lowest to serotype 3 (PV3) [3]. In 2009, the baseline seroprevalence of antibodies to PV3 among infants aged 6–9 months was just 48% in a community-based randomised clinical trial conducted in a high risk area, Moradabad in northern India [12].

We now report a community-based seroprevalence study of anti-poliovirus type 3 antibodies among infants of age 5–11 months who had not previously received inactivated poliovirus vaccination (IPV) residing in rural and urban areas of Vellore district of Tamil Nadu, southern India. This study was done to screen for a clinical trial on the effect of azithromycin on the immunogenicity of serotype-3 monovalent oral poliovirus vaccine (mOPV3) given to healthy infants without antibodies to serotype-3 poliovirus [10], which found that removal of bacterial pathogens by azithromycin treatment did not increase the proportion of children who responded to mOPV3.

## 2 Methods

### 2.1 Study design and setting

The cross sectional survey was carried out in 210 health sub-centres (HSC, each serves a population of 5000) of 42 primary health centres (PHC, each serving approximately 20,000–30,000) in 14 health blocks (serving 80,000–120,000 and as referral facility for 3–4 PHCs) of the rural and urban parts of Vellore district of Tamil Nadu between July 2014 and January 2015.

Infants in the study area receive routine immunisation either from government or private health care facilities, tOPV is given with BCG at birth and at 6, 10 and 14 weeks along with DPT in the study area. IPV was not available in the government sector during the study period. The last supplemental immunisation activity (SIA) was in February 2014 and no SIAs were carried out during the study period.

The Christian Medical College Institutional Review Board and the Imperial College Research Ethics Committee approved the study and appropriate central and state governmental permissions were obtained prior to conducting the screening. Investigators and study coordinators met with local community leaders, private and government health providers and informed them about the study and requested their cooperation.

### 2.2 Study population

We did a door to door survey to identify infants and written informed consent was obtained from all willing parents of eligible healthy infants aged between 5 and 11 months. The Village Health Nurses (VHNs) of the concerned Health sub-centres discussed the study with potential participants and motivated the families to participate. A screening camp was organised in each village, and parents brought the child to the camp. Each infant was assigned a unique screening identification number and basic demographic details were collected. A study physician examined the infant for eligibility for screening and recorded the infant’s age and polio vaccination history from the immunisation cards. Additional doses received during National Immunization Days were obtained from verbal history as these doses are not recorded on immunisation cards and if immunisation cards were not available, the mother’s statement was recorded. Exclusion criteria included children who had received IPV-through private healthcare providers, had any congenital or chronic illness or had high grade fever or any other illness that prevented participation as decided by the study physician. Infants temporarily excluded because of minor illnesses were asked to visit the camp held in a neighbouring village.

### 2.3 Laboratory methods

Blood specimens were collected by trained phlebotomists and study nurses. Samples were stored on ice and delivered to the laboratory on the same day. Assessment of poliovirus-specific neutralising antibodies to serotype 3 was done using a micro-neutralisation assay according to the World Health Organization (WHO) protocol with modifications [13]. Briefly, a 2-fold dilution of each serum sample (50 μl) ranging from 1/4 to 1/8 was mixed with 50 μl of approximately 100 median tissue culture infective dose (TCID_50_) of Sabin 3 poliovirus in replicate wells at each dilution and the mixture was incubated at 37 °C (5% CO_2_) for 1 h. 100 μl of Vero cell suspension (5000 cells/well) was then added to all the wells and the plates were incubated for 3 days at 37 °C (5% CO_2_). As part of quality control, standard polio antisera from the U.S. Centers for Disease Control and Prevention were included in each run. For each assay, a back-titration titre of 30–300 TCID_50_ was considered acceptable. Cell controls were included in each assay. A reciprocal titre of <8 was considered non-protective.

### 2.4 Statistical methods

#### 2.4.1 Data management and analysis

Single data entry of the questionnaire was done using Epi-Info 3.5.1 (CDC, GA, USA) and was verified by a statistician. SPSS 18 (SPSS Inc., IL, USA) and STATA 13 (StataCorp, TX, USA) software were used for analysis. All statistical tests were carried out at 5% significance level (p < 0.05) and confidence interval (CI) set at 95%. Student’s *t*-test was used to compare means. Univariable and multivariable logistic regression was performed on sex, age, number of OPV doses received and place of residence and odds ratio (with 95% CI) calculated to ascertain the strength of association between the exposure and outcome variable. In addition, multilevel logistic regression (MLR) was performed to examine the effect of contextual (location, period, administrative) and compositional (individual variation) effects on model parameter estimates [14]. We estimated the probability of seroconversion for each dose of OPV using the binomial likelihood function, allowing for an arbitrary baseline level of seroprevalence among infants who were reported to have received OPV.

## 3 Results

### 3.1 Baseline characteristics

Of 12609 children in the target age group invited to participate; 429 (3%) refused participation, 1224 (10%) had received IPV, 2150 (17%) did not visit camp sites because they were unlikely to be available and 352 (3%) were screen failures based on medical grounds (congenital heart disease, fever, etc.). Samples were collected from 8454 children from 10 rural health blocks and 4 urban health centres with an average of 604 children per block (range 89–1184), with a mean age of 8.3 (SD-1.8) months. Baseline characteristics of the study population are shown in Table 2.

### 3.2 Seroprevalence

**Fig. 1:**
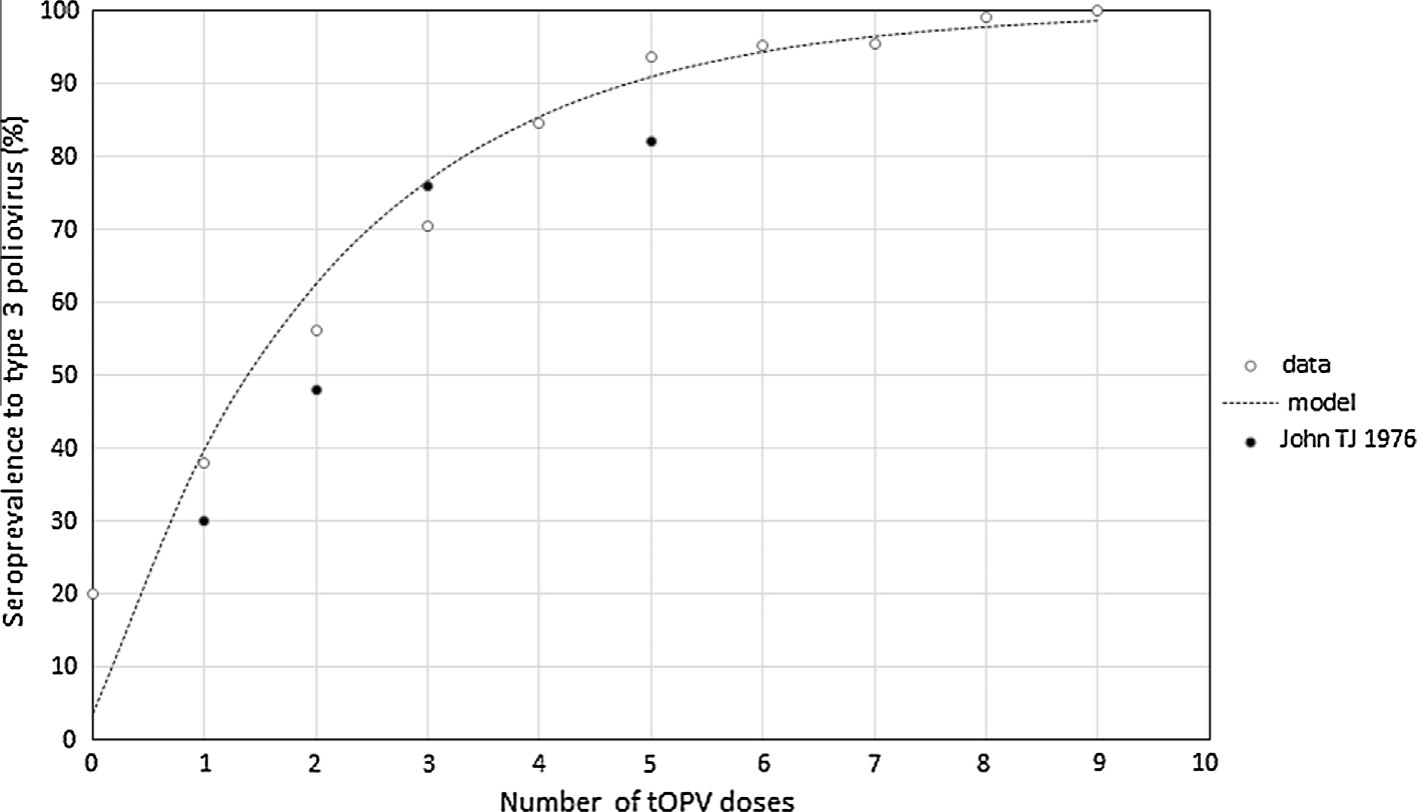
Seroprevalence of anti-poliovirus type 3 antibodies by number of OPV doses compared with binomial maximum likelihood model. Estimates reported by John TJ 1976 [16] from the same area are shown by way of comparison.

The overall seroprevalence of PV3 antibodies was 88.1% (95% CI: 87.4–88.8), 89.2% (95% CI: 88.3–90.1) for males and 87.0% (95% CI: 85.9–88.0) among females (p - <0.0001). Of the 8449 subjects for whom immunisation status was recorded (median, 4 doses; interquartile range [IQR], 4–6 doses), children who received fewer doses had lower seroprevalence, with the lowest 20% (95% CI: 0.5–71.6) among children who reported receiving no dose. The estimated probability of seroconversion with each reported dose of OPV was 37.7% (95% CI: 36.7–38.3), with an estimated baseline seroprevalence of 3.6% (95% CI: 3.2–4.0) (Fig. 1).

**Fig. 2:**
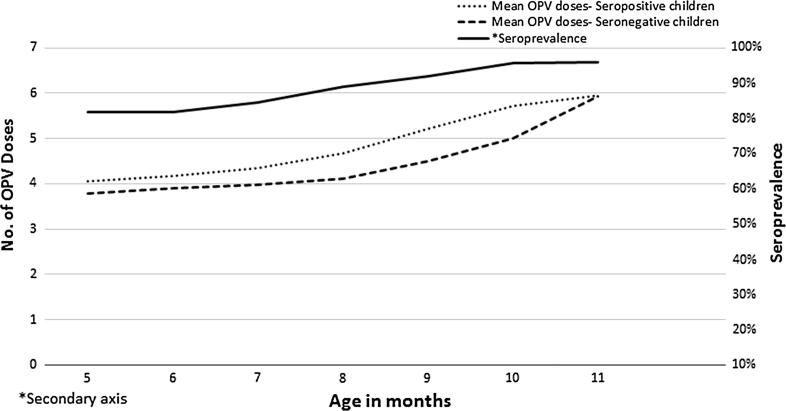
Seroprevalence of anti-poliovirus type 3 antibodies by mean number of OPV doses and age among orally immunised Indian infants.

The mean age of seropositive children was higher than seronegative children (8.4 months, 95% CI: 7.4–7.6 vs. 7.5 months, 95% CI: 8.4–8.5, p < 0.001, *t*-test). When stratified by month of age, 5-month old children had the lowest seroprevalence 81.8% (95% CI: 79.0–84.3), seroprevalence increased with age and was 95.9% (95% CI: 94.2–97.3) for the 11 month olds. Chi-square (χ^2^) value for linear trends (extended Mantel Haenszel test) for age and number of OPV doses were 232.5 (*p* < 0.001) and 336.8 (*p* < 0.001) respectively. PV3 seropositivity trend analysis for mean age and mean number of OPV doses is shown in Fig. 2.

There was no difference in seroprevalence between rural (5552, 87.8%) and urban (1899, 89.3%) children in the univariable analysis (*p* = 0.059). Seroprevalence was different across the health blocks, ranging from 82.1% (95% CI: 79.2–84.8) to 92.55% (95% CI: 87.3–96.1), as listed in Table 3. Comparison of standard and MLR of null model revealed significant unexplained heterogeneity (likelihood-ratio χ^2^ = 40.57 with 1 degree of freedom; p = <0.001) across blocks.

### 3.3 Univariable and multivariable regression

The results of the univariable, multivariable and multivariable mixed effects logistic regression analysis are presented in Table 4. In the multivariable regression, male children were more likely to be seropositive (OR 1.27, 95% CI: 1.11–1.46). In this same model, children had higher odds for PV3 seropositivity for each month increase in age (OR 1.17, 95% CI: 1.12–1.23) and one dose increase of OPV (OR 1.74, 95% CI: 1.61–1.89). Similarly children residing in urban areas had higher odds of being seropositive compared to rural children (OR 1.24, 95% CI: 1.05–1.45) in the standard multivariable regression analysis but this was not significant when analysed in the multivariable mixed effects logistic regression at the block level (OR 1.18, 95% CI: 0.98–1.41). The mixed effects regression performed to investigate the cluster effect at various levels show a significant random effects at the block level (likelihood-ratio χ^2^ = 11.3 with 1 degree of freedom; p = 0.0004) and not at the PHC and HSC levels. However, we did not find any appreciable changes in the parameter estimates.

## 4 Discussion

The seroprevalence among infants of age 5–11 months in rural and urban areas of Vellore district of Tamil Nadu who had not previously received IPV was 88.1% (95% CI: 87.4–88.8). This is similar to rates in Pakistan (Table 1), but slightly lower than in Sri Lanka [15].

The seroprevalence increased with number of OPV doses received and is comparable to the seroconversion rates reported by John [16] and in concurrence with Indian [17], [18], [19] and other studies [20], [21], [22], [23]. Similar to other studies, seroprevalence rates increased with age [19], [21], [24], [25]. This could be due to a more mature immune system or to receipt of OPV doses that are not recorded in the vaccination history taken for the child.

Interestingly, there was limited variability in seroprevalence rates across the various rural and urban blocks (Table 3), demonstrating that the Tamil Nadu’s state immunisation system is able to substantially deliver vaccines.

Our study demonstrated that urban infants have higher seroprevalence which may be due to increased coverage, accessibility and higher literacy rates. This is similar to a Mexican study where urban children had higher antibody prevalence rates (88.2%) than rural children (82.9%) [23] but differs from other studies that report no difference by place of residence [26], [27].

Seroprevalence was marginally higher (89.2%) in males compared to females (87.0%) consistent with another study [22], but differing from Nigeria where females had higher antibody titres for all the three serotypes in one study [28] or were not different [29] and a Chinese study that also found no difference [30]. Sex differential non-specific effects of vaccines are common in developing countries with negative non-specific effects (NSE) of inactivated vaccines more common in girls than boys [31], [32] in some countries, but with no differences in high income countries [33]. Though the general pattern is both negative and positive NSE are stronger in females [34], a randomised controlled trial in Guinea-Bissau negated the hypothesis that mortality rates in boys would be lower if they had not received OPV0 [35]. The reasons why OPV uptake is slightly higher for males in our study area are unknown.

Though factors such as age, gender and urban setting have no remedial solution from a public health perspective, this study indicates that response may be affected by factors that are not amenable to modification. This is important for the understanding of oral vaccine performance in low-income countries.

## 5 Limitation

One of the limitations of this study was that we were unable to collect more detailed demographic information, morbidity and nutritional status, other than the physician’s assessment because of logistic constraints.

## 6 Conclusion

To our knowledge this is the largest seroprevalence study in a low income setting in a narrow age range. Seroprevalence was associated with age, gender, number of OPV doses received and place of residence. The high rate of seropositivity to the weakest antigen in the trivalent OPV in recent studies in India differs markedly from older studies where low rates of seropositivity to this antigen were reported. Perhaps the recent switch from tOPV to bOPV will further increase immunogenicity to type 3 by elimination of the interference by type 2.

India has seen improvements in immunisation coverage such that seroprevalence is now reasonable. However, immunogenicity remains lower than in high-income countries, consistent with results for other oral vaccines. For future oral vaccines, understanding the reasons for poor performance in low-income settings remains important.

## Authors’ contributions

All authors participated in the design or implementation or analysis, or interpretation of the study; and the development of this manuscript. The work described was carried out in accordance to ICMJE recommendations for conduct, reporting, editing and publication of scholarly work in medical journals. The corresponding author had final responsibility to submit for publication.

GK, JM, NG and JJ designed the study; SV AKS collected and assembled data; IP, SG and SB carried out the laboratory works, SPK, SV and NG analysed study data; GK and NG interpreted study data, SPK wrote the first draft, GK and NG edited the drafts. All the authors had full access to the data and gave final approval before submission.

## Funding

This work was supported by the Bill & Melinda Gates Foundation, Seattle, WA [OPP1039135].

## Role of the funding source

The funders had no role in the design of the study; collection, analysis and interpretation of data; writing of the report; or the decision to submit the paper for publication. The corresponding author had full access to all the data from the study and had final responsibility for the decision to submit for publication.

## Conflict of interest

The authors declare no conflict of interest.

## Figures and Tables

**Table 1:** Type specific seroprevalence of anti-poliovirus antibodies among infants in lower and upper middle income countries.

Place	World Bank Income classification	Year of study	Age group	Sample size	Sero prevalence			References
					Type 1 (%)	Type 2 (%)	Type 3 (%)	
Egypt	Lower middle income	2004	6–11 months	973	99.0	99.0	91.0	[36]
Moradabad, India	Lower middle income	2007	6–12 months	467	88.0	70.0	75.0	[19]
Islamic republic of Iran	Upper middle income	2010	7 months	72	84.7	95.8	70.8	[37]
Kano, Northern Nigeria	Lower middle income	2011	6–9 months	161	81.0	75.0	73.0	[22]
Northern India	Lower middle income	2010	6–7 months	1280	98.0	66.0	77.0	[38]
Pakistan	Lower middle income	Published in 2013	6–11 months	554	96.0	87.9	86.7	[39]
Sri Lanka	Lower middle income	2014	9–11 months	100	96.0	98.0	85.0	[15]

**Table 2:** Baseline characteristic of the study population.

Characteristic	Number of children screened	Percentage seroprevalence for PV3 (95% CI)	
Total	8454	88.1	(87.4–88.8)
*Age, months*			
5	862	81.8	(79.0–84.3)
6	1633	81.9	(79.9–83.7)
7	1453	84.5	(82.5–86.3)
8	1312	89.0	(87.2–90.7)
9	1222	92.1	(90.4–93.5)
10	1261	95.7	(94.5–96.8)
11	711	95.9	(94.2–97.3)

*Sex*			
Male	4366	89.2	(88.3–90.1)
Female	4088	87.0	(85.9–88.0)

*Area*			
Urban	2127	89.3	(87.9–90.6)
Rural	6327	87.8	(86.9–88.6)

CI-confidence interval.

**Table 3:** Seroprevalence of anti-poliovirus type 3 antibodies by health block and mean number of oral poliovirus vaccine doses received among the study population.

Health block name	Seroprevalence (%)	Number of screened children	Mean number of OPV doses
Alamelumangapuram	149(92.6)	161	5.1
Ambedkarnagar	77(86.5)	89	4.6
Anaicut	1061(89.6)	1184	4.9
Arcot	935(89.5)	1045	4.7
Kaniyambadi	751(91.8)	818	5.0
Kaspa	196(88.7)	221	4.3
Katpadi	488(89.1)	548	4.8
Kaveripakkam	735(84.0)	875	4.4
Lakshmipuram	190(92.2)	206	4.8
Nemili	611(82.1)	744	4.3
Ranipet	171(87.7)	195	4.5
Thimiri	502(84.5)	594	4.9
Ussoor	822(87.7)	937	4.9
Walajah	763(91.2)	837	4.7
Overall	7451(88.1)	8454	

OPV-oral poliovirus vaccine.

**Table 4:** Factors associated with seroprevalence of anti-poliovirus type 3 antibodies by univariable, multivariable and multilevel multivariable logistic regression analysis among orally immunised Indian infants (N = 8454 for Unadjusted values except No. of OPV doses; N = 8449 for adjusted values since OPV status not available for 5 children).

Characteristic	Standard univariable		Standard multivariable		Multilevel multivariable					
					HSC		PHC		Block	
	Odds Ratio (95% CI)	*p* value	Odds Ratio (95% CI)	*p* value	Odds Ratio (95% CI)	*p* value	Odds Ratio (95% CI)	*p* value	Odds Ratio (95% CI)	*p* value
Sex Male	1.24 (1.08–1.41)	0.002	1.27 (1.11–1.46)	0.001	1.27 (1.11–1.46)	0.001	1.27 (1.11–1.46)	0.001	1.27 (1.11–1.45)	0.001
Age in months	1.37 (1.31–1.43)	<0.001	1.17 (1.12–1.23)	<0.001	1.17 (1.12–1.23)	<0.001	1.17 (1.12–1.23)	<0.001	1.17 (1.12–1.23)	<0.001
No. of tOPV doses	1.94 (1.80–2.09)	<0.001	1.74 (1.61–1.89)	<0.001	1.74 (1.60–1.89)	<0.001	1.73 (1.59–1.88)	<0.001	1.72 (1.58–1.87)	<0.001
Area Urban	1.16 (0.99–1.36)	0.059	1.24 (1.05 –1.45)	0.010	1.24 (1.05– 1.46)	0.012	1.25 (1.05– 1.49)	0.013	1.18 (0.98–1.41)	0.087
Constant			0.0969128		0.0970927		0.0971074		0.1109489	
−2Log Likelihood			−2853.48		−2853.45		−2851.90		−2847.82	
Chibar2(01)					0.06		3.16		11.33	
Prob of Chibar2 > 0					0.4012		0.0376		0.0004	

CI – Confidence Interval HSC – Health Sub-centre PHC – Primary Health Centre.
